# Protein expression profiles and clinicopathologic characteristics associate with gastric cancer survival

**DOI:** 10.1186/s40659-019-0249-0

**Published:** 2019-08-09

**Authors:** Wei Li, Yan Chen, Xuan Sun, Jupeng Yang, David Y. Zhang, Daguang Wang, Jian Suo

**Affiliations:** 1 Department of Gastrointestinal Surgery, The First Hospital of Jilin University, Changchun, 130021 Jilin China; 2 Jilin Province Key Laboratory of Bioinformatics for Gastrointestinal Tumor, Changchun, Jilin China; 3 Department of Pathology, Mount Sinai School of Medicine, New York, NY USA

**Keywords:** Gastric cancer, Protein expression profiling, Tissue microarray, Immunohistochemistry, Pathway

## Abstract

**Background:**

Prognosis remains one of most crucial determinants of gastric cancer (GC) treatment, but current methods do not predict prognosis accurately. Identification of additional biomarkers is urgently required to identify patients at risk of poor prognoses.

**Methods:**

Tissue microarrays were used to measure expression of nine GC-associated proteins in GC tissue and normal gastric tissue samples. Hierarchical cluster analysis of microarray data and feature selection for factors associated with survival were performed. Based on these data, prognostic scoring models were established to predict clinical outcomes. Finally, ingenuity pathway analysis (IPA) was used to identify a biological GC network.

**Results:**

Eight proteins were upregulated in GC tissues versus normal gastric tissues. Hierarchical cluster analysis and feature selection showed that overall survival was worse in cyclin dependent kinase (CDK)2, Akt1, X-linked inhibitor of apoptosis protein (XIAP), Notch4, and phosphorylated (p)-protein kinase C (PKC) α/β2 immunopositive patients than in patients that were immunonegative for these proteins. Risk score models based on these five proteins and clinicopathological characteristics were established to determine prognoses of GC patients. These proteins were found to be involved in cancer related-signaling pathways and upstream regulators were identified.

**Conclusion:**

This study identified proteins that can be used as clinical biomarkers and established a risk score model based on these proteins and clinicopathological characteristics to assess GC prognosis.

## Background

Gastric cancer (GC), an epithelial malignancy, is the third leading cause of cancer deaths globally, accounting for 7% of cancer cases and 9% of cancer deaths globally [1]. According to China’s Cancer Statistics, GC is the second most common malignancy in China [2]. It is difficult to cure unless it is detected at an early stage. However, because early GC patients present with few symptoms, the cancer is usually at an advanced stage when diagnosed [3]. Presently, GC treatments include surgical resection, chemotherapy, and/or radiation therapy [4, 5]. Surgery together with chemotherapy or other therapy methods has been shown to be much more effective than surgery alone [6]. Nevertheless, the prognosis of GC is poor due to metastasis. The 5-year survival rate for advanced GC is less than 10% [7, 8]; therefore early diagnosis is vital for successful treatment.

Despite advances in biotechnology and endoscopy screening, more than 80% of patients with GC are diagnosed at an advanced stage or experience tumor recurrence after surgery [9], which significantly affects prognosis. Existing histopathologic classification schemes, including Lauren classification [10], Ming classification [11], Goseki classification [12], and World Health Organization histologic type and grade classification, cannot predict a patient’s prognosis [13]. There is an urgent need for additional biomarkers to identify patients at risk of poor outcomes or recurrence.

GC is a heterogeneous disease, and its initiation and progression are influenced strongly by genetic and environmental factors [14]. Presently, many candidate gene products, such as MUC1, CEA, p53, p16, and E-cadherin, have been suggested to predict the survival of patients with GC [15–19]. Although there has been much investigation of the genetic factors that predict survival, few genetic alterations have been used in GC diagnosis. Recently, it has become possible to conduct large-scale molecular studies on formalin-fixed tissue samples with tissue microarrays and immunohistochemistry. Such large-scale studies have involved numerous markers and cases. For example, a large-scale cluster analysis showed that multiple markers correlated significantly with patient survival [20, 21]. Therefore, tissue microarrays and immunohistochemistry may be a practical method for routine testing and validation studies.

In a previous study, we screened for GC-associated signaling proteins using protein pathway arrays (PPAs) and found that 22 proteins, or phosphorylated (p)-protein forms, were differentially expressed between cancer and normal tissues. Of those 22 proteins, the following 9 were upregulated in GC tissues [1]: proliferating cell nuclear antigen (PCNA), Notch4, cyclin-dependent kinase (CDK)4, CDK6, X-linked inhibitor of apoptosis protein (XIAP), p-protein kinase C (PKC)α/βII, Akt, β-catenin, and p-PKCα. In this study, we aimed to verify overexpression of these proteins in GC using tissue microarrays and immunohistochemistry. We also analyzed survival characteristics to establish prognostic scoring models to improve clinical outcome predictions.

## Materials and methods

### Patients and tissue samples

This study included 121 surgically resected primary GC samples collected from patients who underwent D2 gastrectomy over a period of 5 years, between January of 2006 and December of 2012, and 30 normal gastric samples from patients who underwent gastrectomy for non-cancer diseases. Two pathologists confirmed the histological diagnoses and tumor-node-metastasis (TNM) staging of the collected tissues. Clinicopathological characteristics of the GC patients, including gender, age, tumor size, tumor location, histologic differentiation, vascular or lymphatic invasion, and tumor stage, were obtained by reviewing medical charts and pathology records (Table 1). None of the patients received preoperative chemotherapy or radiotherapy. Patients were followed-up from the date of surgery for a period of 6–119 months (mean, 55 months) and clinical outcomes were recorded. Survival time was calculated from the date of surgery to the date of death or the last day of follow-up. The majority of the patients died of the cancer. Cases lost to follow-up were not included in our survival analysis. The Institution Ethical Review Board of The First Hospital of Jilin University approved this study, and all of the patients provided informed consent.

**Table 1:** Summary of patient demographics and clinicopathological characteristics

Clinicopathological characteristics	Patients N = 121 (%)
Age (years)	
≤ 60	57 (47.1)
> 60	64 (52.9)
Gender	
Male	91 (75.2)
Female	30 (24.8)
Tumor location	
Lower third	85 (70.2)
Middle third	23 (19.0)
Upper third	13 (10.8)
Tumor size (cm)^a^	
≤ 5	94 (77.7)
> 5	27 (22.3)
Histologic differentiation	
Moderate	38 (31.4)
Poor	83 (68.6)
Vascular/lymphatic invasion	
Absent	37 (30.6)
Present	84 (69.4)
T stage	
T1	7 (5.8)
T2	29 (24.0)
T3	84 (69.4)
T4	1 (0.8)
N stage	
N0	35 (29.0)
N1	45 (37.1)
N2	29 (24.0)
N3	12 (9.9)
TNM stage^b^	
I	19 (15.7)
II	27 (22.4)
III	62 (51.2)
IV	13 (10.7)

^a^Tumor size measured in greatest transverse diameter (cm)

^b^TNM staging was performed according to the American Joint Committee on Cancer. No patient had metastasis to distant organs (M0)

### Tissue microarrays

Tissue microarrays were prepared as previously described [22]. Briefly, whole sections of individual donor tissue blocks, which were stained with hematoxylin and eosin (H&E), were used to select tumor areas for tissue microarray cores. Three cylinders of tissues (0.6 mm in diameter) were punched from each sample and re-embedded in a recipient paraffin block at predetermined positions. Multiple 4-µm-thick sections were cut from each tissue array block and mounted on microscope slides.

### Immunohistochemistry

Sections on tissue microarray slides were dewaxed in xylene and then rehydrated in a series of graded alcohols. Antigen retrieval was performed by autoclaving the sections in citrate buffer (pH 6.0) for 2 min and then cooling them in dH_2_O. Then, the sections were immersed in 3% hydrogen peroxide in phosphate-buffered saline (PBS) for 15 min to block endogenous peroxidase activity. Non-specific binding was then blocked in 10% normal goat serum at room temperature for 10 min. Subsequently, the sections were incubated with primary antibodies (Table 2) at 4 °C overnight in a moist chamber. After washing with PBS, the sections were incubated with secondary antibodies for 1 h at room temperature. The sections were stained with 3,3-diaminobenzidine and counterstained with Harris hematoxylin, dehydrated, and mounted. Immunoreactivity was evaluated microscopically by two pathologists. Protein staining was graded according to a previous study [23]: 0, negative, − (no cells stained); 1, weakly positive, + (< 10% of cells stained); 2, moderately positive < ++ (10–50% of cells stained); or 3, strongly positive, +++ (> 50% cells stained).

**Table 2:** Antibodies used for immunohistochemistry

Antibody	Dilution	Clonality	Source
p-PKCα	1:100	Monoclonal	Abcam
p-PKCα/β2	1:50	Polyclonal	Abcam
Akt1	1:100	Polyclonal	Abcam
CDK6	1:200	Polyclonal	Santa Cruz
Notch4	1:200	Polyclonal	Santa Cruz
β-Catenin	1:50	Polyclonal	GenScript
CDK2	1:50	Polyclonal	Invitrogen
PCNA	1:200	Monoclonal	BioLegend
XIAP	1:200	Polyclonal	BioLegend

### Cluster analysis

Hierarchical cluster analysis was conducted in the Cluster program (complete linkage clustering) [24]. Clustering analysis results were displayed in TreeView software [25]. Expression data were graded as follows: − 3, negative staining; 1, weak positive staining; 2, moderately positive staining; and 3, strongly positive staining.

### Signaling network analysis

To visualize the interactions and upstream regulators of differentially expressed proteins, pathway and network analyses were carried out in Ingenuity Pathway Analysis version 9.0 (IPA), a protein-gene and protein–protein interaction analysis program.

### Statistical analysis

Chi-square test and Fisher’s exact (two-sided) tests were used to determine associations between protein expression status and clinicopathological variables. Kaplan–Meier survival curves were created, and differences between the curves were examined by log-rank testing. Cox proportional hazard regression analysis was used to determine independent prognostic factors. Principal component analysis (PCA) was performed to establish a survival prediction model for GC patients. Additionally, the Kaiser–Meyer–Olkin measure and Bartlett’s test of sphericity were used to ensure appropriate extraction factor analysis. To determine the optimal prognosis prediction model, receiver operating characteristic (ROC) curve analysis was applied to principal components (PCs), and the area under the curves (AUCs) were calculated. All analyses were performed in SPSS 17.0 (SPSS Inc, Chicago, IL). A p value < 0.05 was considered to be statistically significant.

## Results

### Protein expression profiling in GC and normal tissues

**Fig. 1:**
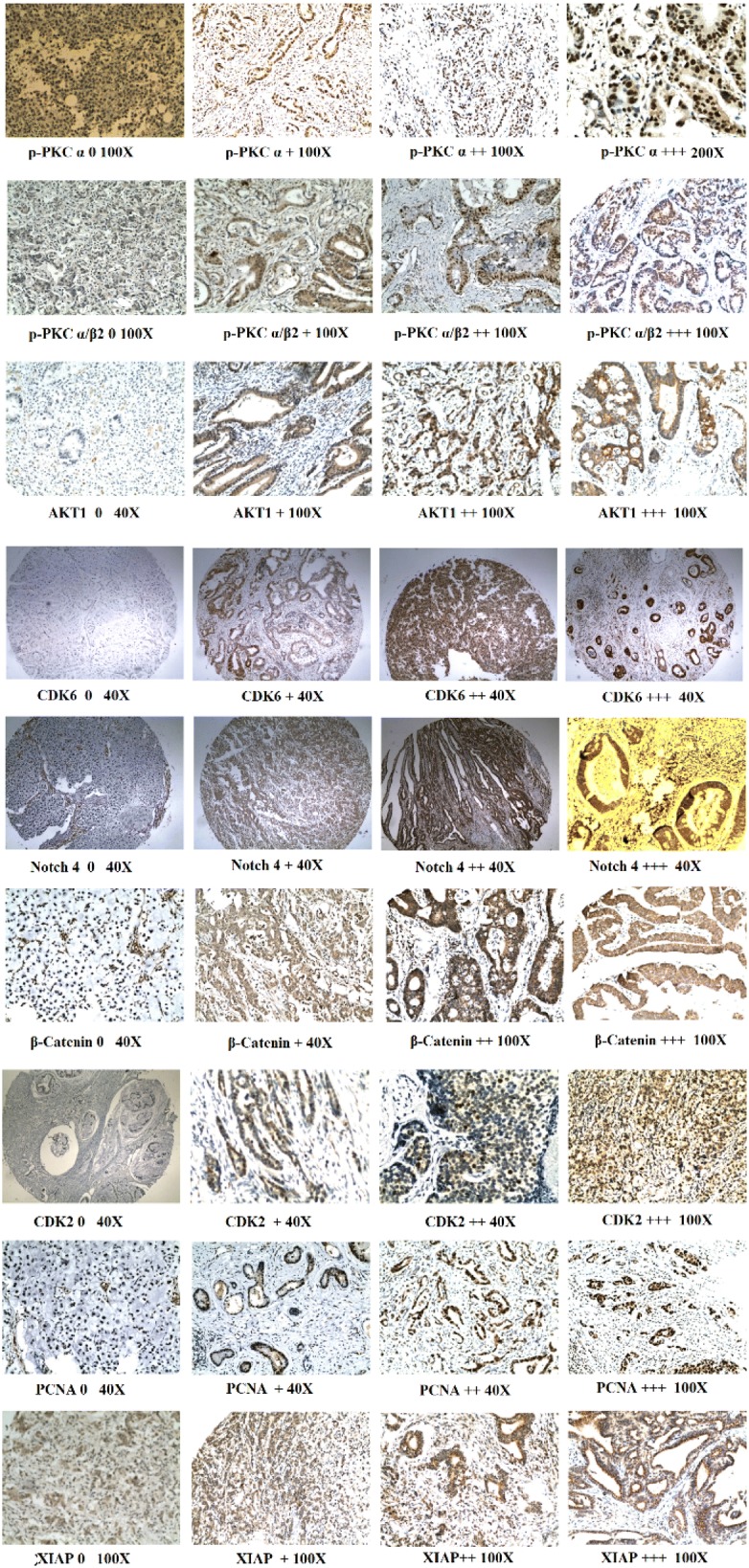
Protein expression detected by immunohistochemistry

The immunohistochemistry results for the nine evaluated proteins are shown in Fig. 1 and summarized in Table 3. Notably, p-PKCα was expressed in 97.5% (117/121) of the GC cases, with weakly positive, moderately positive, and strongly positive expression in 46, 59, and 12 cases, respectively. Meanwhile, p-PKCα was expressed in 66.7% (20/30) of the normal tissues, with weakly positive, moderately positive, and strongly positive expression in 10, 19, 1 and 0 cases, respectively. p-PKCα/β2 was expressed in 82.6% (100/121) of the GC cases; weakly positive, moderately positive, and strongly positive expression was seen in 59, 37, and 4 cases, respectively, whereas weakly positive p-PKCα/β2 expression was observed in 32.5% (13/30) of the normal tissues. Akt1 was expressed in 81.8% (99/121) of the GC cases, and weakly positive, moderately positive, and strongly positive expression was seen in 73, 20, and 6 cases, respectively, whereas weakly positive Akt1 expression was seen in 33.3% (10/30) of the normal tissues. CDK6 was expressed in 62.0% (75/121) of the GC cases, and weakly positive, moderately positive, and strongly positive expression was seen in 68, 4, and 4 cases, respectively, whereas weakly positive CDK6 expression was seen in 83.3% (25/30) of the normal tissues. Notch4 was expressed in 95.0% (115/121) of the GC cases, with weakly positive, moderately positive, and strongly positive expression in 64, 48, and 3 cases, respectively, whereas weakly positive Notch4 expression was seen in 73.3% (22/30) of the normal tissues. β-Catenin was expressed in 96.7% (117/121) of the GC cases, with weakly positive, moderately positive, and strongly positive expression in 96, 21, and 0 cases, respectively. β-Catenin was expressed in 93.3% (28/30) of the normal tissues, with weakly positive, moderately positive, and strongly positive expression in 27, 1, and 0 cases, respectively. CDK2 was expressed in 56.2% (68/121) of the GC cases, with weakly positive, moderately positive, and strongly positive expressio in 55 cases, 12 cases, and 1 case, respectively, whereas no CDK2 immunopositivity was seen in normal tissues. PCNA was expressed in 97.5% (118/121) of the GC cases, and weakly positive, moderately positive, and strongly positive expression in 7, 57, and 54 cases, respectively. PCNA was expressed in 73.3% (22/30) of the normal tissues, with weakly positive, moderately positive, and strongly positive expression in 21, 1, and 0 cases, respectively. XIAP was expressed in 97.5% (101/121) of the GC cases, with weakly positive, moderately positive, and strongly positive expression in 63, 32, and 6 cases, respectively. XIAP was expressed in 93.3% (28/30) of the normal tissues, with weakly positive, moderately positive, and strongly positive expression in 26, 2, and 0 cases, respectively. In GC cells, p-PKCα/β2, Akt1, CDK6, Notch4, and PCNA were expressed mainly in the nucleus and the cytoplasm. CDK6 was also expressed in some muscle tissues near GC cells. p-PKCα, CDK2, and XIAP were expressed mainly in the nucleus with low-level expression in the cytoplasm. Of the nine proteins, eight (p-PKCα, p-PKCα/β2, Akt1, CDK6, Notch4, CDK2, PCNA, and XIAP) were upregulated in GC tissues when compared to normal tissues (Chi-square or Fisher’s exact tests, p < 0.05; Table 3).

**Table 3:** Expression of nine GC-associated proteins in GC and normal tissues detected by immunohistochemistry

Protein	GC (N = 121) No. (%) of patients				Normal gastric tissues (N = 30) No. (%) of patients				p value
	−	+	++	+++	−	+	++	+++	
p-PKCα	4 (3.3)	46 (38.0)	59 (48.8)	12 (9.9)	10 (33.3)	19 (63.3)	1 (3.3)	0 (0)	< *0.001*
p-PKCα/β2	21 (17.4)	59 (48.8)	37 (30.6)	4 (3.3)	17 (56.7)	13 (43.3)	0 (0)	0 (0)	*<0.001* ^#^
Akt1	22 (18.2)	73 (60.3)	20 (16.5)	6 (5.0)	20 (66.7)	10 (33.3)	0 (0)	0 (0)	*<* *0.001*
CDK6	46 (38.0)	68 (56.2)	4 (3.3)	3 (2.5)	5 (17.7)	25 (83.3	0 (0)	0 (0)	*0.022* ^#^
Notch4	6 (5.0)	64 (52.9)	48 (39.7)	3 (2.5)	8 (26.7)	22 (73.3)	0 (0)	0 (0)	*<* *0.001*^#^
β-Catenin	4 (3.3)	96 (79.3)	21 (17.4)	0 (0)	2 (6.7)	27 (90.0)	1 (3.3)	0 (0)	0.119
CDK2	53 (43.8)	55 (45.5)	12 (9.9)	1 (0.8)	30 (100)	0 (0)	0 (0)	0 (0)	*<* *0.001*^#^
PCNA	3 (2.5)	7 (5.8)	57 (47.1)	54 (44.6)	8 (26.7)	21 (70)	1 (3.3)	0 (0)	*<* *0.001*
XIAP	20 (16.5)	63 (52.1)	32 (26.4)	6 (5.0)	2 (6.1)	26 (78.8)	2 (6.1)	0 (0)	0.007

Data are shown for negative (−), weakly positive (+), moderately positive (++), and highly positive (+++) labeling

p values were obtained by Chi-square test or the Fisher’s exact test; ^#^Fisher’s exact test used with theoretical frequency < 1, italic values: p < 0.05

### Correlations between protein expression profiles and clinicopathologic parameters of GC

Correlations between protein expression status (negative vs. positive) and clinicopathologic characteristics were determined (summarized in Table 4). Positive p-PKCα/β2 and CDK2 expression in primary GC was associated with older age (p < 0.05). Increased CDK2 expression also correlated with the presence of vascular/lymphatic invasion (p = 0.014), advanced N stage (p = 0.042), and advanced TNM stage (p = 0.020).

**Table 4:** Correlation between protein expression and clinicopathologic characteristics (p values are shown)

Variables	p-PKCα	p-PKCα/β2	Akt1	CDK6	Notch4	CDK2	PCNA	XIAP
Age (years)	> 0.99	0.048	0.213	0.211	0.160	0.001	0.919	0.836
Gender	0.256^#^	0.659	0.804	0.489	0.338	0.628	0.573^#^	0.163
Tumor location	0.217^#^	0.404	0.092	> 0.99	0.288^#^	0.453	0.372^#^	> 0.99
Tumor size	0.574^#^	0.494	0.173	0.570	> 0.99	0.066	0.535^#^	0.983
Histologic differentiation	0.407	0.758	0.332	0.56	0.212	0.516	0.551^#^	0.882
Vascular/lymphatic invasion	> 0.99	0.459	0.889	0.098	> 0.99	0.014	0.552^#^	0.553
T stage	> 0.99^#^	0.586^#^	0.111^#^	0.701^#^	0.356^#^	0.123^#^	> 0.99^#^	0.925^#^
N stage	0.790^#^	0.745	0.504	0.103	0.390^#^	0.042	0.236^#^	0.575
TNM stage	0.766^#^	0.714	0.390	0.447	0.647^#^	0.020	> 0.99^#^	0.765

p values were obtained by Chi-square or Fisher’s exact tests; ^#^p values obtained by Fisher’s exact test used with theoretical frequency < 1

### Hierarchical cluster analysis of GC and survival-associated feature selection

**Fig. 2:**
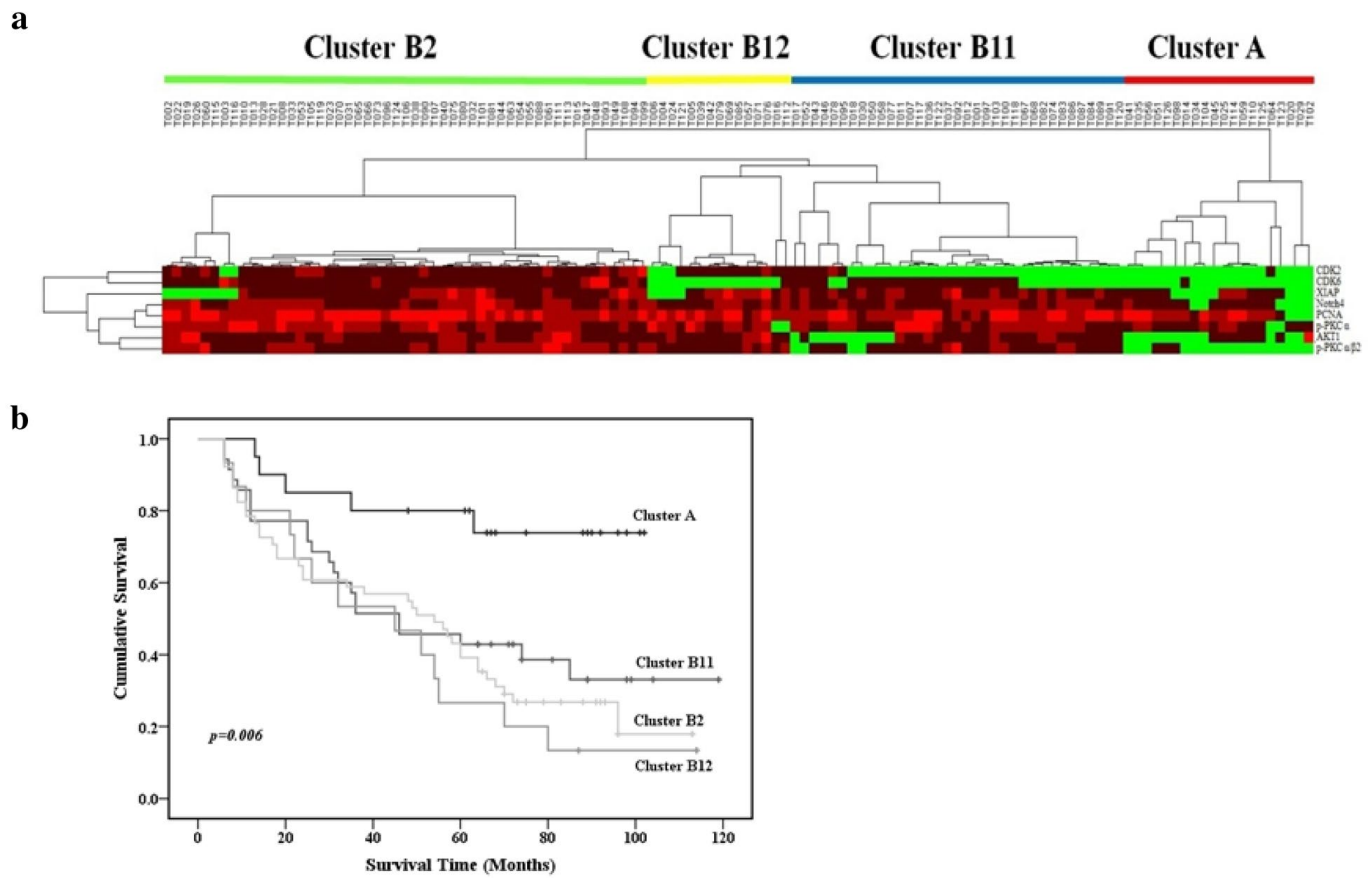
Classification of 121 GCs based on the expression of eight proteins. **a** A data matrix in which each row corresponds to a single protein biomarker and each column corresponds to a single tumor. Colors represent expression levels: negative expression (green) and positive expression (red). Horizontal bars correspond to clusters. **b** Univariate Kaplan–Meier survival analysis. p values were determined by log-rank testing

Hierarchical cluster analysis was performed with eight protein expression profiles from 121 GC cases. Tumors were separated into two clusters based on protein expression patterns (Fig. 2a). Cluster A contained 20 cases and Cluster B contained 101 cases. Eight of the proteins had much lower (including negative) expression in Cluster A than in Cluster B. Furthermore, Cluster B was subdivided into three subclusters: B11 (35 cases), B12 (15 cases), and B2 (51 cases). Comparison of differences in clinicopathological characteristics among the four (sub)clusters, showed that Cluster A contained earlier TNM-stage cases than the three B subclusters. Cluster B2 contained more cases with vascular/lymphatic invasion than Cluster A. Kaplan–Meier analysis revealed that Cluster A cases had better survival than cases in Clusters B11, B12, and B2 (p = 0.006; Fig. 2b). Multivariate Cox proportional hazard regression analysis of age, tumor size, histologic differentiation, vascular/lymphatic invasion, TNM stage, and expression of the eight proteins (Cluster A vs. Cluster B) showed that expression of the eight proteins was an independent prognostic indicator of survival (hazard ratio, 5.822; 95% confidence interval, 2.317–14.625; p < 0.001). In addition, age at surgery, tumor size, and TNM stage were also independent prognostic indicators of survival with hazard ratios of 1.914 (95% confidence interval, 1.186–3.089; p = 0.008), 1.989 (95% confidence interval, 1.175–3.367; p = 0.01), and 1.961 (95% confidence interval, 1.294–2.971; p = 0.002), respectively.

**Fig. 3:**
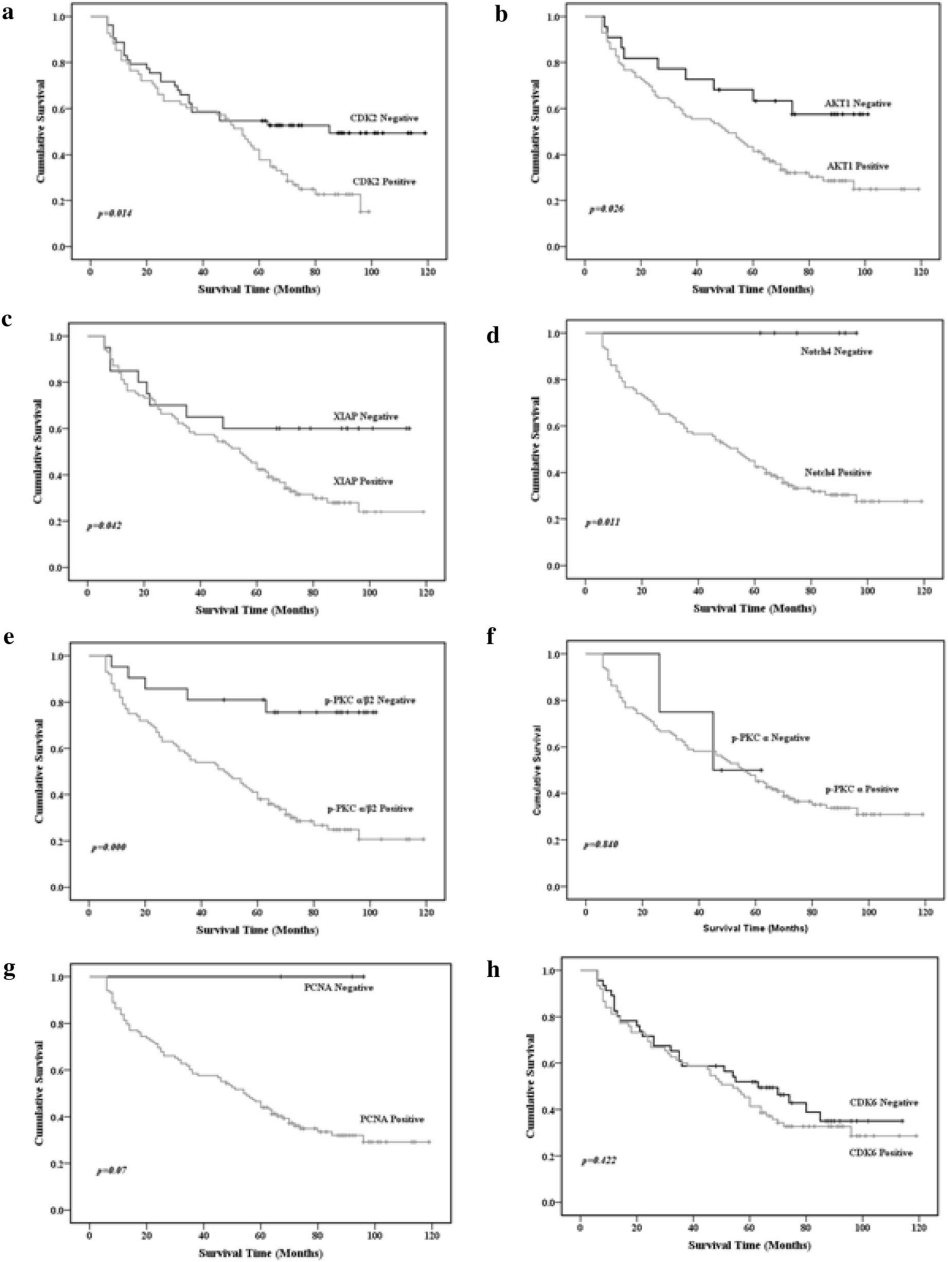
Expression of **a** CDK2, **b** Akt1, **c** XIAP, **d** Notch4, **e** p-PKCα/β2, **f** p-PKCα, **g** PCNA and **h** CDK6 and their association with overall survival. The five proteins with p < 0.05 were chosen by feature selection using the Kaplan–Meier method. p values were determined by log-rank testing

Feature selection was performed with the Kaplan–Meier method (univariate analysis) to identify protein expression profiles associated with survival. Overall survival was worse in cases with CDK2, Akt1, XIAP, Notch4, and p-PKCα/β2 than in cases without immunopositivity for these proteins (log-rank test: p = 0.014, 0.026, 0.042, 0.011, and < 0.001, respectively; Fig. 3).

**Fig. 4:**
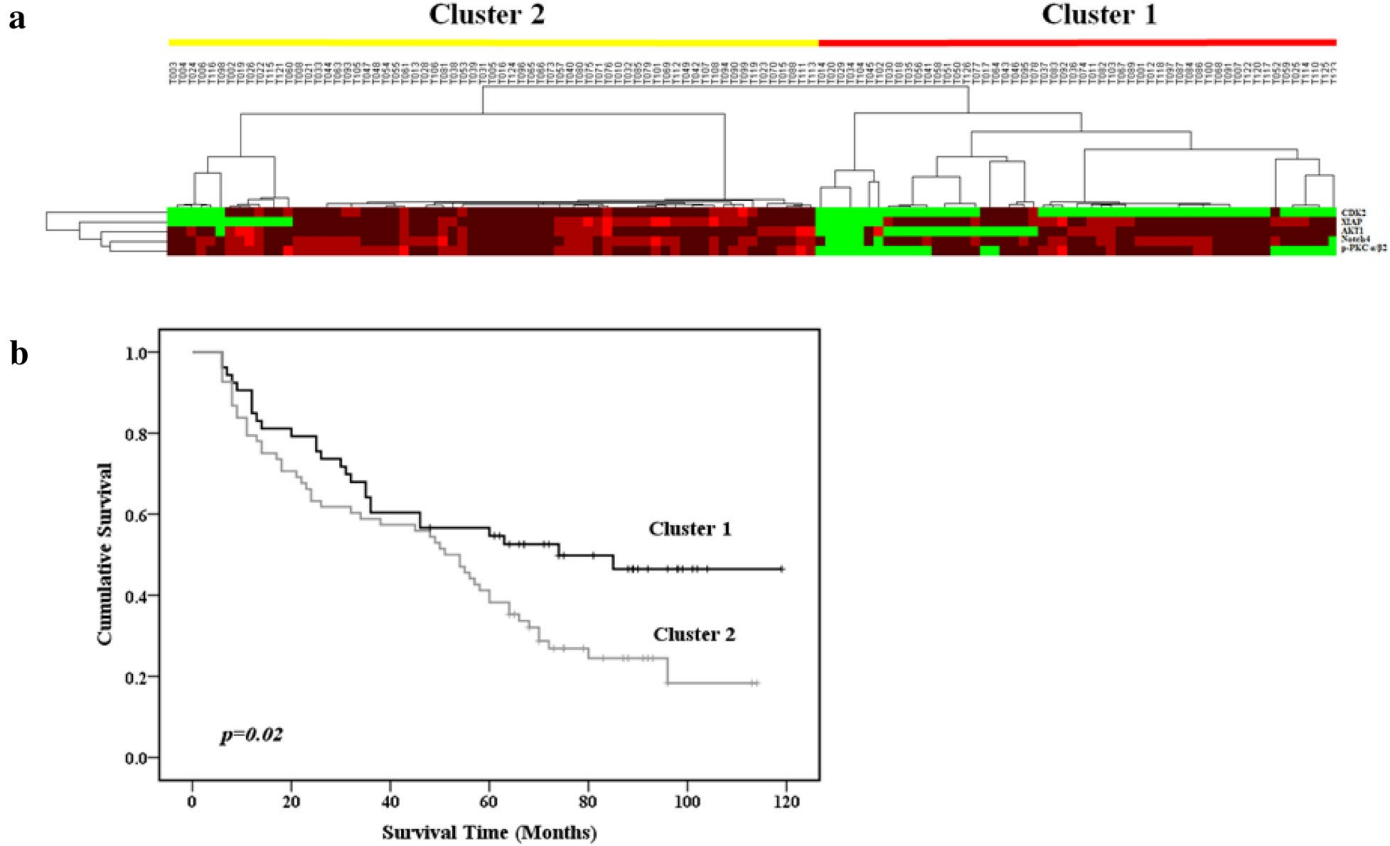
Classification of 121 GC samples based on expression of five survival-associated proteins after feature selection. **a** A matrix representing the data and showing two GC subgroups: Clusters 1 and 2. **b** Univariate Kaplan–Meier survival analysis. p values were determined by log-rank testing

After feature selection, further hierarchical cluster analysis of 121 cases and five survival-associated proteins (CDK2, Akt1, XIAP, Notch4, and p-PKCα/β2) segregated the cases into Cluster 1 (53 cases) and Cluster 2 (68 cases) (Fig. 4a). Univariate analysis performed to determine whether these clusters were indicative of clinically distinct subgroups showed that Cluster 2 cases had poorer prognoses than Cluster 1 cases (p = 0.02; Fig. 4b). Multivariate analysis revealed that this classification (Cluster 1 or 2) was an independent prognostic indicator of survival (hazard ratio, 2.370; 95% confidence interval, 1.447–3.879; p = 0.001).

### Establishment of a risk model to predict prognoses of GC patients

**Fig. 5:**
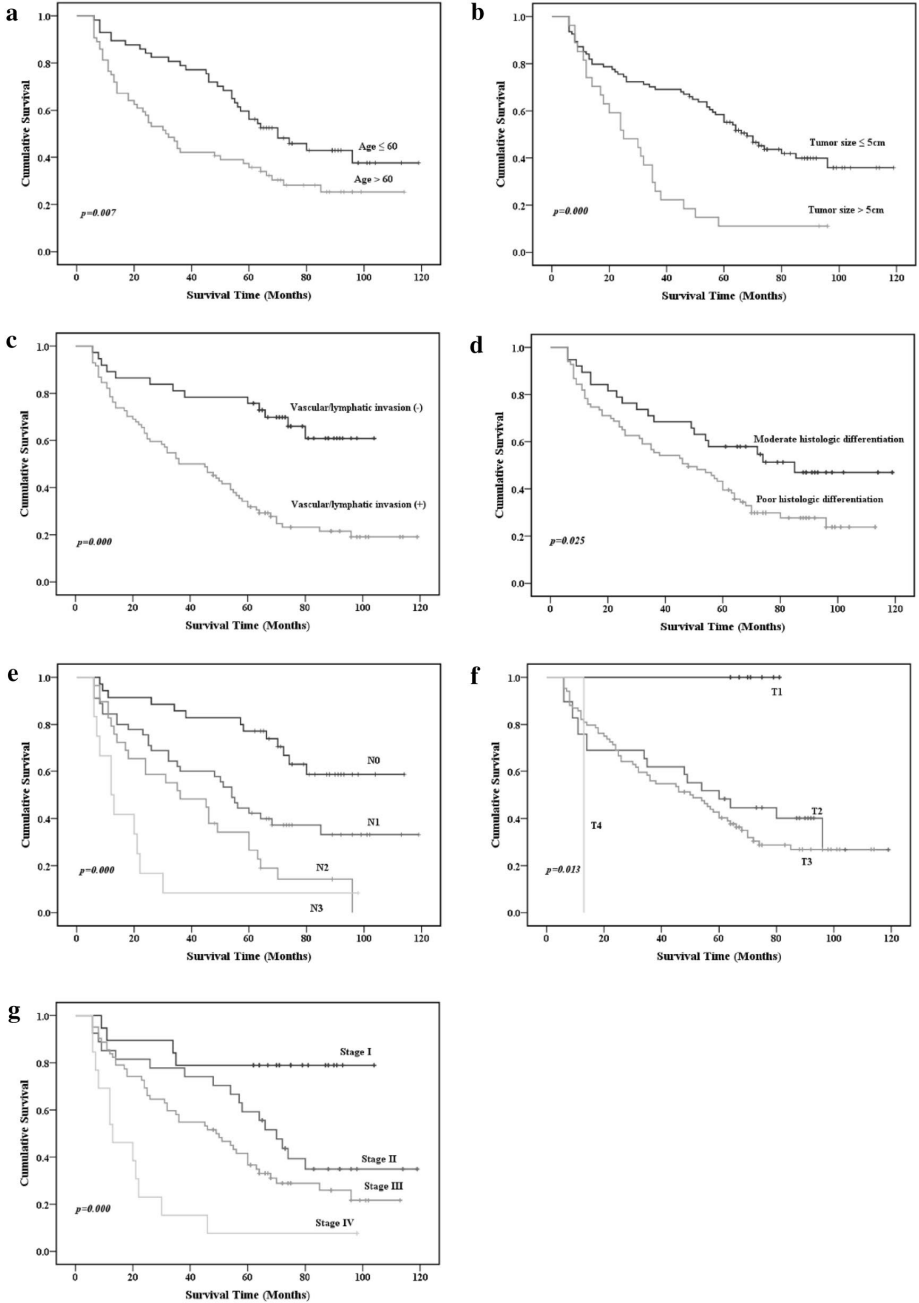
Univariate Kaplan–Meier analysis of clinicopathological variables associated with the prognosis of GC patients, including **a** Age at surgery, **b** tumor size, **c** Vascular/lymphatic invasion, **d** Histologic differentiation, **e** N stage, **f** T stage and **g** TNM stage. p values were determined by log-rank testing

Kaplan–Meier analysis (univariate) performed to identify clinicopathological variables associated with GC prognosis indicated that age at surgery, tumor size, T stage, N stage, TNM stage, vascular/lymphatic invasion, and histologic differentiation correlated with overall survival (p< 0.05; Fig. 5). To exclude redundant variables, five proteins (CDK2, AKT1, XIAP, Notch4, and p-PKC α/β2) and five clinicopathological variables (age of surgery, tumor size, TNM stage, vascular/lymphatic invasion, and histologic differentiation) were included in a PCA analysis to extract features. T stage and N stage were excluded from the analysis because they are encompassed in the TNM stage variable. The proteins and clinicopathological variables were considered categorical covariates with corresponding categories and codes: protein expression (immunonegative = 0, immunopositive = 1); gender (female = 0, male = 1); age (≤ 60 = 0, > 60 = 1); tumor size (≤ 5 = 0, > 5 = 1); histologic differentiation (moderate = 0, poor = 1); vascular/lymphatic invasion (absent = 0, present = 1); TNM stage (I = 0, II = 1, III = 2, IV = 3). The code “0” was assigned to the reference category. The Kaiser–Meyer–Olkin measure of sampling adequacy was 0.602 and the significance level (p value) for Barlett’s Test of Sphericity was < 0.001, thus the data were deemed suitable for analysis.

Four factors (PC 1–4) arose from our analysis of PCs with eigenvalues > 1.0 in the PCA. The contributing rate of the cumulative sums of the squares was 65.16%. PC loadings for each of the variables are shown in Table 5. PC 1 was heavily loaded with the Notch4, p-PKCα/β2, XIAP, DK2, and Akt1 variables and termed the protein factor. PC 2 was heavily loaded with the TNM stage and vascular/lymphatic invasion variables and was termed the pathological factor. PC 3 was heavily loaded with the age and tumor size variables and was termed the clinical factor. PC 4 was heavily loaded with the histologic differentiation variable. PC scores (calculated in SPSS) indicated that each of these four factors was independent of the others.

**Table 5:** Component matrix of variables associated with gastric cancer prognosis

Variables	Principal components (PC)			
	1	2	3	4
Notch4	*0.798*	0.128	00.019	− 0.200
p-PKC α/β2	*0.726*	− 0.071	0.353	0.107
XIAP	*0.652*	0.048	− 0.371	− 0.128
CDK2	*0.563*	− 0.408	0.088	0.279
AKT1	*0.532*	− 0.059	0.448	− 0.013
TNM staging	0.029	*0.902*	− 0.069	0.095
Vascular/lymphatic invasion	− 0.013	*0.873*	0.047	0.134
Age	0.101	− 0.103	*0.680*	0.065
Tumor size	− 0.060	0.383	*0.598*	− 0.324
Histologic differentiation	− 0.096	0.210	− 0.020	*0.867*

Extration methods: Principal components analysis. Italic valus: the variables in different PCs

**Fig. 6:**
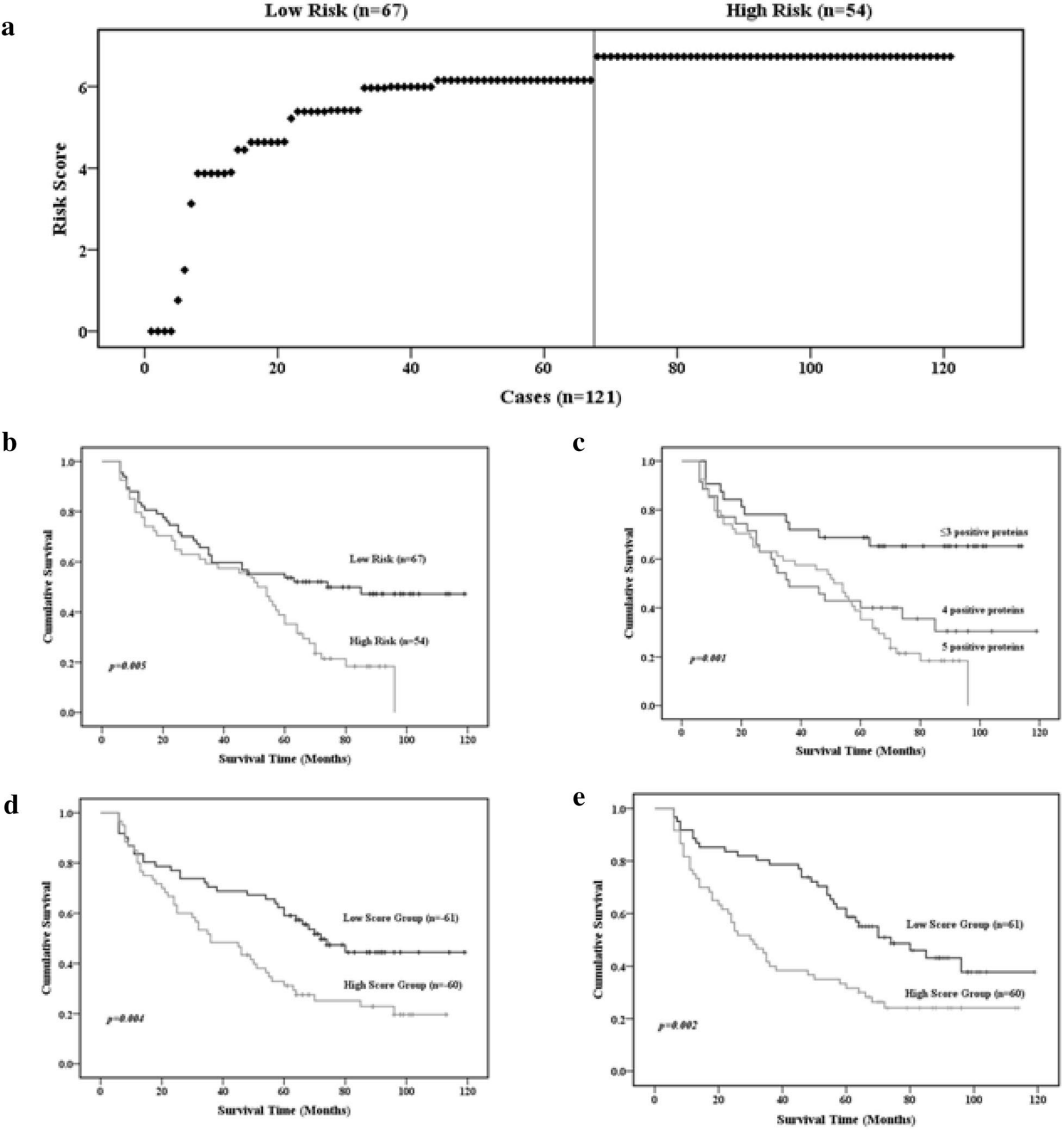
Kaplan–Meier survival analysis of patients with GC and risk score models. **a** A risk score model based on expression of five proteins. The patients were ranked according to their risk scores; the line divides the patients into low-risk and high-risk score groups. **b** Kaplan–Meier survival analysis of patients in the low-risk and high-risk score groups based on the five-protein risk score model. **c** Kaplan–Meier survival analysis of patients with different numbers of immunopositive proteins. **d** Kaplan–Meier survival analysis of patients in the low-risk and high-risk score groups based on TNM stage and vascular/lymphatic invasion. **e** Kaplan–Meier survival analysis of patients in the low-risk and high-risk score groups based on tumor size and age. p values were determined by log-rank testing

Because PC 1 was a five-protein factor, we established a risk score model based on the five proteins to predict GC prognosis. Risk scores were calculated on the basis of protein expression status (immunonegative/immunopositive) and the corresponding regression coefficients from univariate Cox proportion hazard regression analysis. Risk scores for patients were calculated by multiplying the regression coefficient of a protein by the protein expression status for each protein and then summing the values. The regression coefficients for Notch4, p-PKCα/β2, XIAP, CDK2, and Akt1 were 3.13, 1.52, 0.74, 0.58, and 0.77, respectively. Therefore, in this study, a risk score = 3.13 × (Notch4) + 1.52 × (p-PKCα/β2) + 0.74 × (XIAP) + 0.58 × (CDK2) + 0.77 × (Akt1). The distribution of risk scores for the 121 patients (range, 0–6.73) is presented in Fig. 6a. Based on the risk score curve, the patients were separated into two groups: low-risk (scores < 6.15; 54 cases) and high-risk (scores ≥ 6.15; 67 cases). Kaplan–Meier analysis revealed that overall survival of patients was worse in the high-risk score group than in the low-risk score group (log-rank test: p = 0.005; Fig. 6b) indicating that the risk score model based on Notch4, p-PKCα/β2, XIAP, CDK2, and Akt1 expression predicts GC prognosis.

To further determine whether the number of immunopositive proteins indicated differential overall survival, we regrouped the patients according to numbers of immunopositive proteins: five immunopositive proteins (6.73; 54 cases), four immunopositive proteins (5.96–6.15; 35 cases), and ≤ 3 immunopositive proteins (0–5.41; 32 cases). Kaplan–Meier analysis showed that patients with ≤ 3 immunopositive proteins had better overall survival (log-rank test: p = 0.001), whereas patients with 4 or 5 immunopositive proteins had similar and worse overall survival (Fig. 6c).

TNM stage and vascular/lymphatic invasion were categorical covariates. To improve the prognosis predicting efficiency of PC 2 (TNM stage and vascular/lymphatic invasion), we separated PC scores in PC 2 by median score. Kaplan–Meier analysis indicated that the low-score group (≤ median) had better overall survival than the high-score group (> median) (log-rank test: p = 0.004; Fig. 6d). The PC scores in PC 3 (age and tumor size) were also separated by median score. Kaplan–Meier analysis revealed that the low-score group had better overall survival than the high-score group (log-rank test: p = 0.002; Fig. 6e). Thus, PC 2 and PC 3 scores can predict GC prognosis. Histologic differentiation in PC 4 separated GC patients into two natural groups. Patients with poor histologic differentiation had worse overall survival than those with moderate differentiation.

**Fig. 7:**
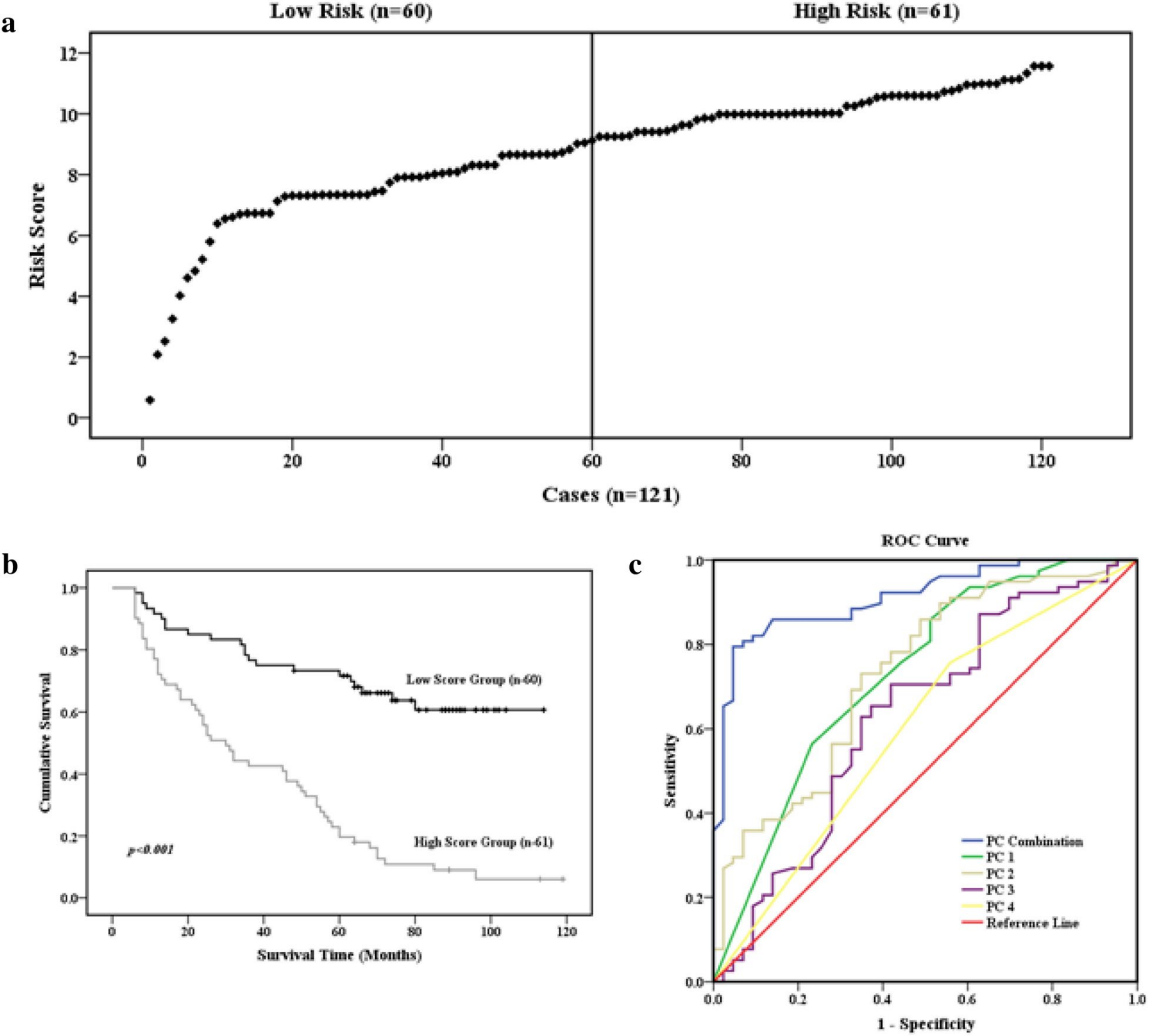
Association between the PC combination risk score model and survival. **a** The PC combination risk score model was based on five proteins and five clinicopathological variables from four PCs. The patients were ranked according to their risk scores; the line divides patients into low-risk and high-risk score groups. **b** Kaplan–Meier survival analysis of patients in the low-risk and high-risk score groups. p values were determined by the log-rank test. **c** ROC curves for the various prognosis prediction models. Five prediction models, PC combination, PC1, PC2, PC3, and PC4, were included in the analysis. The PC combination and PC1 models were better predictors than the other models (p < 0.05)

ROC curve analysis AUCs for PCs 1–4 were 0.728, 0.732, 0.630, and 0.599, respectively. The sensitivity/specificity/accuracy of PCs 1–4 were 81.48%/49.2%/63.64%, 76.67%/47.54%/61.98%, 75%/45.90%/60.33%, and 71.08%/50%/64.465, respectively. To obtain a risk score prognostic model with higher sensitivity, specificity, and accuracy, a risk score model (PC combination) based on the five proteins and the five clinicopathological variables from the four PCs was established. The risk score was calculated as follows: risk score = 3.13 × (Notch4) ± 1.52 × (p-PKCα/β2) + 0.74 × (XIAP) + 0.58 × (CDK2) + 0.77 × (Akt1) + 0.74 × (TNM stage) + 1.20 × (vascular/lymphatic invasion) + 0.97 × (tumor size) + 0.61 × (age) + 0.58 × (histologic differentiation). The distribution of risk scores for the 121 patients (range, 0.58–11.57) is presented in Fig. 7a. Based on the median score (9.25), the patients were separated into low-score (< 9.25; 60 cases) and high-score (≥ 9.25; 61 cases) groups. Kaplan–Meier analysis revealed that overall survival was worse in the high-score group than in the low-score group (log-rank test: p = 0.005; Fig. 7b). The AUC, sensitivity, specificity, and accuracy were 0.912, 96.72%, 70%, and 83.47% for the PC combination risk model, and these values are higher than the values obtained for the four individual PCs (p < 0.001). Thus, the PC combination risk score model predicted the prognosis of GC patients better than the four PCs (Fig. 7c).

### Identification of signaling networks associated with survival of patients with GC

**Fig. 8:**
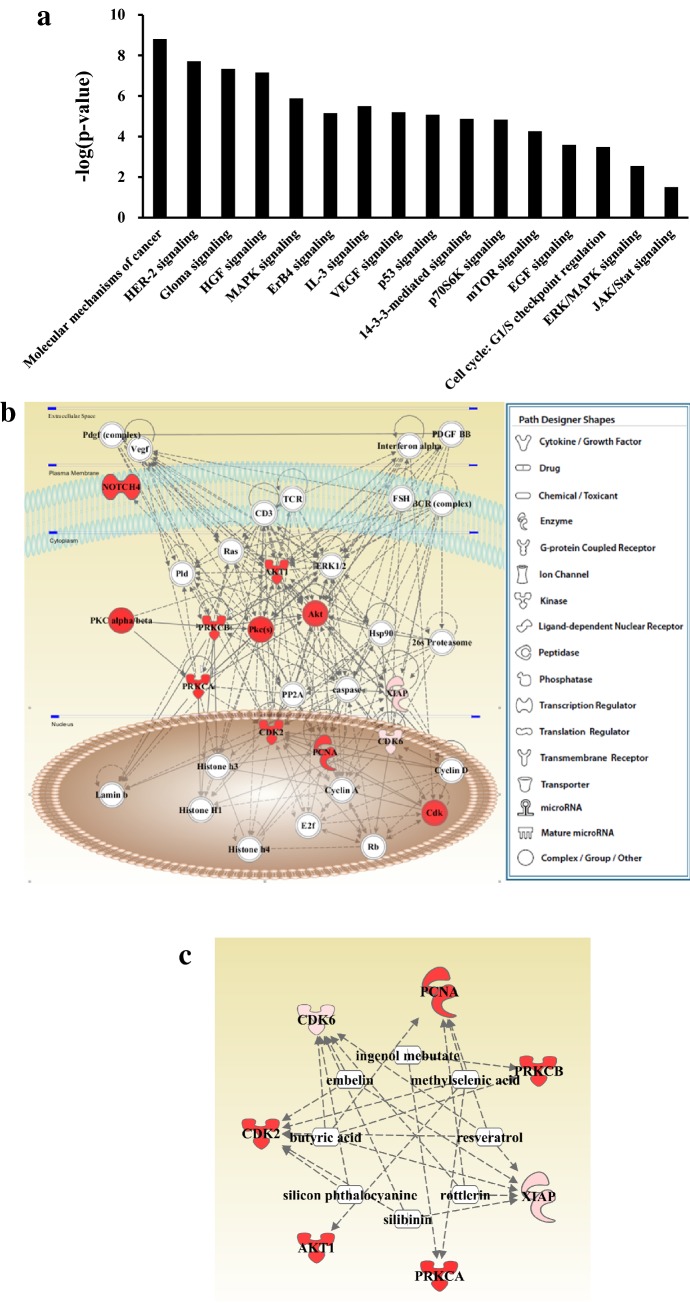
Signaling networks associated with GC. **a** The top canonical pathways (identified by IPA) that were associated with the eight evaluated proteins are shown. The −log p values indicate the significance of the signaling pathways based on the number of differentially expressed proteins involved. **b** IPA-generated signaling pathway network with a score of 23. **c** Upstream analysis of the pathway. Proteins whose expression was detected are indicated with red color and the depth of color indicates the degree of expression. Different shapes indicate different functions

Pathway analysis revealed that the proteins analyzed in this study were involved in pathways related to cellular survival and death (eight proteins; p = 1.34 × 10^−9^–1.84 × 10^−2^), the cell cycle (seven proteins; p = 7.40 × 10^−9^–1.66 × 10^−2^), cell development (eight proteins; p = 8.33 × 10^−9^–1.58 × 10^−2^), cell growth and proliferation (eight proteins; p = 8.33 × 10^−9^–1.58 × 10^−2^), and DNA replication, recombination, and repair (four proteins; p = 1.02 × 10^−6^–1.66 × 10^−2^). Additionally, pathway analysis in the diseases and disorders category showed that all eight of the proteins studied were related to cancer (p = 3.00 × 10^−8^–1.80 × 10^−2^), and seven of the proteins associated with metabolic disease (p = 6.47 × 10^−8^–1.10 × 10^−2^). IPA results revealed that the eight proteins evaluated in this study are involved in several canonical signaling pathways, including molecular mechanisms of cancer (p = 1.56 × 10^−9^), HER-2 signaling (p = 1.97 × 10^−8^), gloma signaling (p = 4.66 × 10^−8^), HGF signaling (p = 7.01 × 10^−8^), MAPK signaling (p = 1.31 × 10^−6^), ErB4 signaling (p = 7.02 × 10^−6^), IL-3 signaling (p = 3.17 × 10^−6^), VEGF signaling (p = 6.28 × 10^−6^), p53 signaling (p = 8.4 × 10^−6^), 14-3-3-mediated signaling (p = 1.35 × 10^−5^), p70S6K signaling (p = 1.47 × 10^−5^), mTOR signaling (p = 5.45 × 10^−5^), EGF signaling (p = 2.59 × 10^−4^), cell cycle G1/S checkpoint regulation (p = 3.28 × 10^−4^), ERK/MAPK signaling (p = 2.81 × 10^−3^), and JAK/Stat signaling (p = 3.13 × 10^−2^; Fig. 8a).

Two networks with scores of 23 and 0 were identified by IPA; the former was selected for further analysis. In this network, Akt, CDK2, PKC, caspase, ERK1/2, and cyclin D interacted closely with additional protein signals (Fig. 8b). Upstream analysis of the IPA network detected 525 upstream regulators of the eight proteins, including plant extracts, chemical medicines, microRNAs, transcriptional regulators, kinases, and cytokines. The most significant upstream regulators included embelin (CDK2, CDK6, XIAP; p = 4.73 × 10^−9^), butyric acid (CDK2, CDK6, PCNA, PRKCB, XIAP; p = 1.06 × 10^−7^), silibinin (CDK2, CDK6, XIAP; p = 5.19 × 10^−7^), silicon phthalocyanine (CDK2, CDK6; p = 1.34 × 10^−6^), ingenol mebutate (PRKCA, PRKCB; p = 1.34 × 10^−6^), rottlerin (CDK6, PCNA, XIAP; p = 1.41 × 10^−6^), methylselenic acid (AKT1, CDK2, PCNA, PRKCA; p = 1.41 × 10^−6^), and resveratrol (CDK2, CDK6, PCNA, XIAP; p = 1.48 × 10^−6^). Overall, PCNA, PRKCB, CDK6, CDK2, AKT1, PRKCA, and XIAP were regulated by 4, 2, 6, 5, 1, 2, and 5 upstream regulators, respectively (Fig. 8c).

## Discussion

In this study, to reduce the limitations to clinical application, we used the tissue microarray method on formalin-fixed specimens to evaluate the expression of nine proteins that we identified in a previous PPA study [1] in 121 primary GC tissues and 30 normal gastric tissues. The results showed that all of the nine proteins were expressed in primary GC tissues and eight of the proteins (all except CDK2) were expressed in normal gastric tissues. Additionally, eight of the proteins were upregulated in GC tissues, in accordance with our previous findings [1].

Hierarchical clustering of immunolabeling data from tissue microarrays of 121 formalin-fixed GC samples with nine GC-associated antibodies yielded patient clusters based on protein expression patterns. The patients in Cluster A (20 cases) had better survival than patients in Cluster B11 (35 cases), Cluster B12 (15 cases), and Cluster B2 (51 cases) (p = 0.006).

Feature selection based on Kaplan–Meier survival analysis yielded Cluster 1 (53 cases) and Cluster 2 (68 cases), which had distinct clinicopathologic features and patient outcomes, with Cluster 2 being associated with poorer prognoses than Cluster 1. Examination of subsequently developed risk score models established to predict clinical outcomes indicated that the PC combination prognosis risk model based on five proteins and five clinicopathological variables was clinically relevant and useful for guiding medical treatment. For example, aggressive chemotherapy may be recommended for patients with high-risk scores in this model to improve survival.

We correlated the five proteins obtained from feature selection with clinicopathologic characteristics. Given the generally long delays from tumorigenesis to diagnosis, protein markers would enable earlier diagnoses. Currently, GC is diagnosed based on symptoms, patients’ knowledge of the disease, and the overall medical condition of the patient. Age is also a major factor in GC diagnosis [26]. In our study, the expression levels of p-PKCα/β2 and CDK2 were higher in patients > 60 years old than in younger patients. In addition to age, depth of invasion can be considered a prognostic factor and an indicator of GC progression. Previous studies have shown that vascular/lymphatic invasion status correlates with GC progression [27, 28]. This study showed that upregulation of CDK2 correlates with vascular/lymphatic invasion. CDK2 expression also correlated with N stage and TNM stage, suggesting that CDK2 may play an important role in GC progression, invasion, and metastasis.

Pathway analysis showed that eight of the proteins studied are involved in cellular signaling (p-PKCα and p-PKCα/β2), cell survival and apoptosis (Akt and XIAP), the cell cycle (CDK6 and CDK2), cell differentiation (Notch4), and cell proliferation (PCNA). A deregulated cell cycle is a fundamental aspect of cancer. Normal cells only proliferate in response to mitogenic or developmental signals, whereas cancer cells proliferate unchecked [29]. In addition, upregulation of PCNA, Akt, and CDK2 has been associated with GC [30–32].

Pathway analysis revealed that the proteins evaluated in this study are involved in several canonical signaling pathways, including HER-2 signaling, MAPK signaling, VEGF signaling, and p53 signaling. It has been suggested that HER-2 expression is a prognostic indicator of GC [33]. MAPK signaling mediates many biological events, such as cell proliferation, differentiation, apoptosis, migration, and invasion in various human cancers, including GC [34, 35]. VEGF is a potent angiogenic factor that has been implicated in tumor-induced angiogenesis, which has been shown to be related to GC development and prognosis [36]. p53 is a transcription factor that regulates a complex signal transduction network referred to as the p53 pathway. The p53 tumor suppressor protein plays a critical role in protection from tumor progression by inducing apoptosis or cell cycle arrest [37]. Thus, we speculate that the proteins evaluated in this study are involved in GC progression and prognosis via these pathways.

## Conclusion

In this study, tissues from 121 GC cases were immunolabeled with nine tumor-associated antibodies in tissue microarrays. Hierarchical cluster analysis based on eight upregulated proteins revealed two clusters with different clinicopathologic features and prognoses. Kaplan–Meier method-based feature selection revealed five proteins that correlated strongly with overall survival suggesting that a risk score model including these proteins could predict the prognoses of patients with GC. These proteins have been shown to be involved in cancer-related signaling pathways. Future studies will focus on elucidating the roles of these proteins in GC.

## Data Availability

Not applicable.

## Abbreviations

GC: gastric cancer
PPA: protein pathway array
P: phosphorylated
PCNA: proliferating cell nuclear antigen
CDK: cyclin-dependent kinase
XIAP: X-linked inhibitor of apoptosis protein
PKC: protein kinase C
TNM: tumor-node-metastasis
H&E: hematoxylin and eosin
PBS: phosphate-buffered saline
IPA: ingenuity pathway analysis
PCA: principal component analysis
